# Surgical Site Infection After Abdominal Surgery: Distribution and Determinants From a Cross-Sectional Study

**DOI:** 10.7759/cureus.91124

**Published:** 2025-08-27

**Authors:** Gowthaam Ramesh, Manivannan RJ, Jaykanthan N, Karthikeyan Selvaraj, Suganya P

**Affiliations:** 1 General Surgery, Sree Balaji Medical College and Hospital, Chennai, IND

**Keywords:** abdominal surgery, antibiotic prophylaxis, cross-sectional study, risk factors, surgical site infection, wound infection

## Abstract

Background: Surgical site infections (SSIs) remain one of the most common postoperative complications, particularly following abdominal surgeries, contributing significantly to patient morbidity, prolonged hospitalization, and healthcare costs. Despite advancements in surgical practices, SSI rates remain high in many low- and middle-income countries, including India.

Objectives: This study aimed to estimate the distribution pattern of SSIs following abdominal surgeries and to identify associated risk factors in patients operated on at a tertiary care hospital in Chennai.

Methods: A cross-sectional study was conducted over an 18-month period at Sree Balaji Medical College and Hospital. A total of 70 patients who underwent abdominal surgeries were included based on defined inclusion criteria. Data were collected using a semi-structured proforma, covering demographic, clinical, surgical, and microbiological parameters. SSI was diagnosed based on clinical criteria and confirmed with wound swab cultures when necessary. Statistical analyses included descriptive statistics, chi-square tests, and logistic regression to determine significant associations.

Results: The incidence of SSI in the study population was 22.9%, which was 16 out of 70 patients. SSIs were more common in patients with diabetes mellitus (31.4%), prolonged preoperative hospital stays (> three days), and those who underwent open surgeries (SSI rate: 31%). Dirty wounds had the highest infection rate (60%), while laparoscopic procedures had significantly lower SSI rates (10.7%). Multivariate logistic regression identified prolonged surgery (> two hours), dirty wound classification, preoperative stay > three days, and diabetes as independent predictors of SSI. The most commonly isolated organism was *Escherichia coli*, followed by *Staphylococcus aureus*, with notable resistance to first-line antibiotics.

Conclusion: SSIs following abdominal surgeries are influenced by multiple interrelated factors, many of which are preventable. Identification of high-risk patients, timely prophylactic antibiotic administration, minimally invasive approaches, and strict adherence to aseptic techniques are key to reducing infection rates. The findings from this study highlight the need for targeted infection control strategies and antimicrobial stewardship in Indian surgical settings.

## Introduction

Surgical site infections (SSIs) are a major global postoperative complication, accounting for up to one-third of complications in lower-income countries. Occurring within 30 days (or a year with implants), SSIs cause significant illness, delayed healing, longer hospital stays, and increased mortality [1]. Abdominal surgeries carry a higher risk of SSIs because of potential contamination from the gastrointestinal tract, especially in emergencies. Even with strict sterile techniques, breaching sterile areas increases this risk. Consequently, SSIs are a key indicator of surgical and hospital performance [2,3]. A cross-sectional study in Trichy, Tamil Nadu, revealed that 61.2% of SSIs occurred following abdominal surgeries, with emergency procedures and diabetes being significant contributing factors [4]. In another prospective study conducted in a rural teaching hospital in central India, the cumulative incidence of superficial SSI in abdominal surgeries was as high as 39%, emphasizing the scale of the problem in low-resource settings [5]. Chennai, a major medical hub, faces significant SSI rates, even in well-equipped hospitals performing high-risk procedures like laparotomies. Given the heavy surgical load from across the region, studying specific SSI risk factors in Chennai's unique context is urgently needed.

SSIs exert a dual burden, both clinical and economic. Clinically, they contribute to wound dehiscence, delayed recovery, sepsis, and higher readmission rates. Economically, they lead to increased costs due to prolonged antibiotic use, repeat interventions, additional wound care, and extended hospitalization. A single SSI can increase hospital costs by 20% to 60%, depending on severity and complications [6]. Moreover, in India’s mixed healthcare economy, where out-of-pocket expenditure dominates, SSIs represent a significant socioeconomic burden, particularly for lower-income populations. A study on bacteriological profiles of SSIs in Uttarakhand demonstrated that antibiotic resistance, particularly among *Staphylococcus aureus *and Gram-negative organisms, further complicates treatment and escalates costs [7].

Determinants of surgical site infections

Multiple studies have identified patient-related, surgery-related, and hospital-related risk factors for SSIs:

Patient-Related Factors

Elderly patients and males are more susceptible to SSIs [4]. Patients with comorbidities like diabetes mellitus, anemia, hypoalbuminemia, obesity, and immunosuppression have strong associations with SSIs [5,8]. Poor nutritional status reserves impair wound healing.

Surgery-Related Factors 

Emergency surgeries elevate risk due to higher contamination levels and inadequate preparation [4,6]. Longer duration of procedures (> two hours) correlates with increased infection risk due to greater tissue exposure and fatigue-induced errors [5,9]. Wound classification affects SSI. Clean-contaminated and contaminated wounds show higher SSI prevalence [9].

Hospital-Related Factors

Flaws in sterilization, hand hygiene, and protocol adherence are significant contributors, especially in overburdened public hospitals [10]. Inappropriate selection or timing of prophylactic antibiotics contributes to preventable infections [7]. The aim is to study the distribution pattern and determinants of risk factors for SSI following abdominal surgery in a tertiary care hospital in Chennai.

## Materials and methods

Study design

This study was conducted as a hospital-based cross-sectional study, designed to assess the distribution pattern and determinants of risk factors for SSIs among patients undergoing abdominal surgeries. The cross-sectional nature of the study allowed the collection of real-time clinical data and enabled the identification of associations between potential risk factors and the development of SSIs during the postoperative period.

Study area and setting

The study was carried out at Sree Balaji Medical College and Hospital, a tertiary care teaching hospital located in Chennai, Tamil Nadu. The hospital has a high volume of elective and emergency abdominal surgeries conducted under the Department of General Surgery, thereby providing an appropriate setting for the study of SSIs in a real-world surgical environment.

Study period

The study was conducted over a period of 18 months, from December 2023 to May 2025. This duration was selected to ensure an adequate sample size, account for seasonal variability in infection rates, and facilitate comprehensive patient follow-up.

Study population

The study population comprised adult patients undergoing abdominal surgeries at Sree Balaji Medical College and Hospital during the study period.

Patients who underwent any abdominal surgery, either elective or emergency, and were willing to provide informed consent and comply with postoperative follow-up were included in the study.

Pediatric patients (age < 18 years) and patients not willing to participate or comply with follow-up requirements were excluded from the study.

Sample size calculation

The sample size was calculated using the single population proportion formula:

$n = \frac{Z^2_{\alpha/2} \, P (1-P)}{d^2}$

Where: Z_α/2​_= 1.96 for 95% confidence level, P = 0.122 (estimated proportion of SSIs based on prior studies), d = 0.05 (margin of error). Substituting the values, the minimum required sample size was 65.

Substituting the values: 3.8416*0.122*0.878/0.0025 ≈ 65

To account for a 10% non-response rate, the final target sample size was set at 70 patients.

Sampling technique

A purposive sampling technique was employed, enrolling consecutive eligible patients undergoing abdominal surgeries during the study period, ensuring inclusion until the target sample size was met.

Data collection tool

A semi-structured questionnaire was used to gather information through preoperative assessment interviews, postoperative clinical monitoring, patient records, follow-up visits, and phone calls (Appendix 1).

The questionnaire included 23 key variables grouped as follows:

Dependent Variable

Presence or absence of SSI, defined clinically by redness, swelling, pain at the incision site, discharge or pus, fever (T > 38°C), or gaping wound.

Independent Variables 

Demographics: age, sex, BMI; comorbidities: diabetes mellitus, hypertension, malignancy; lifestyle factors: smoking, alcohol use; surgical details: type of surgery, duration, emergency/elective status; preoperative factors: time and type of shaving, administration of prophylactic antibiotics; postoperative findings: wound characteristics, culture reports, fever.

Antibiotic prophylaxis protocol

All patients received preoperative antibiotic prophylaxis according to institutional protocol. A single dose of intravenous ceftriaxone (1 g) was administered 30-60 minutes before skin incision. In patients with penicillin or cephalosporin allergy, intravenous clindamycin (600 mg) was used. For contaminated or dirty wounds, metronidazole (500 mg IV) was added for anaerobic coverage. Prophylaxis was not routinely extended beyond 24 hours postoperatively, except in selected high-risk patients as determined by the operating surgeon.

Microbiological methods

For suspected SSI, wound swabs were collected under aseptic precautions and transported immediately to the microbiology laboratory. Samples were cultured on blood agar and MacConkey agar and incubated at 37°C for 24-48 hours. Organisms were identified using standard biochemical tests (catalase, coagulase, oxidase, and carbohydrate fermentation profiles). Antimicrobial susceptibility was determined by the Kirby-Bauer disk diffusion method following Clinical and Laboratory Standards Institute (CLSI) guidelines.

Ethical approval and informed consent

This study was conducted after obtaining approval from the Institutional Human Ethics Committee of Sree Balaji Medical College and Hospital (Approval No: 002/SBMCH/IHEC/2023/2065). Written informed consent was obtained from all participants prior to inclusion in the study. Patients were assured of confidentiality, and data were anonymized before analysis.

## Results

The majority of patients undergoing abdominal surgeries were in the 31-40 years age group (25.7%), followed closely by the 41-50 years group (22.9%). The least represented group was patients above 60 years (14.3%). The study sample consisted of more male participants (61.4%) than female participants (38.6%), indicating a male predominance among patients undergoing abdominal surgeries in the study period. Most patients (40%) fell within the normal BMI range. However, nearly one-third (31.4%) were overweight, and one-fifth were obese (20%), indicating that a significant portion of the sample was at potential nutritional or metabolic risk. Diabetes mellitus was the most common comorbidity (31.4%), followed by hypertension (25.7%). Notably, 42.9% of patients had no known comorbidities, highlighting variability in the risk profiles. A significant proportion of patients (30%) had a history of smoking, while 22.9% reported alcohol consumption. These lifestyle factors are important to consider as potential contributors to delayed wound healing and increased SSI risk (Table 1]).

**Table 1:** Demographic characteristics

Variable	n	%
Age (years)		
18–30	12	17.1
31–40	18	25.7
41–50	16	22.9
51–60	14	20
> 60	10	14.3
Gender		
Male	43	61.4
Female	27	38.6
BMI (kg/m²)		
Underweight (< 18.5)	6	8.6
Normal (18.5–24.9)	28	40
Overweight (25.0–29.9)	22	31.4
Obese (≥ 30)	14	20
Comorbidities		
Diabetes mellitus	22	31.4
Hypertension	18	25.7
Malignancy	4	5.7
None	30	42.9
Lifestyle		
Smoking (Yes)	21	30
Smoking (No)	49	70
Alcohol (Yes)	16	22.9
Alcohol (No)	54	77.1

Appendectomy (22.9%) and laparotomy (20.0%) were the most commonly performed abdominal procedures in the study population. A diverse range of surgeries reflects typical case distribution in a tertiary care general surgery department. More than half of the procedures (54.3%) were performed electively. However, a substantial proportion (45.7%) were emergency surgeries, often associated with higher SSI risk due to limited preoperative optimization. The majority of wounds were classified as clean-contaminated (37.1%) and clean (27.1%), but a significant number fell into contaminated and dirty categories, which carry a higher risk for SSI (Table 2).

**Table 2:** Surgical characteristics

Variable	n	%
Type of surgery		
Appendectomy	16	22.9
Laparotomy	14	20
Hernia repair	11	15.7
Cholecystectomy	10	14.3
Bowel resection/anastomosis	8	11.4
Perforation closure	6	8.6
Others	5	7.1
Urgency		
Elective	38	54.3
Emergency	32	45.7
Wound classification		
Clean	19	27.1
Clean-contaminated	26	37.1
Contaminated	15	21.4
Dirty	10	14.3

The SSI rate increased progressively with wound contamination, with the highest incidence observed in dirty wounds (60.0%), followed by contaminated wounds (33.3%). This confirms the strong correlation between wound classification and infection risk. SSI occurrence was significantly higher in surgeries lasting more than two hours (75%), likely due to prolonged tissue exposure, blood loss, and greater bacterial contamination. Shorter procedures showed much lower infection rates. Patients with a preoperative hospital stay of more than three days had a significantly higher SSI incidence (56.3%). Longer stays may increase exposure to hospital-acquired pathogens and indicate sicker patients. SSI rate was substantially higher in patients who did not receive prophylactic antibiotics (50%). Timely administration of prophylaxis plays a crucial role in infection prevention. Patients shaved within two hours prior to surgery had the lowest SSI rate (8.8%), while those shaved more than 24 hours before had a markedly higher rate (40%). This supports current recommendations for just-in-time preoperative hair removal (Table 3).

**Table 3:** Risk factor associations SSI: surgical site infection

Risk factor	SSI present	SSI absent	Total	SSI rate (%)
Wound class - clean	1	18	19	5.3
Clean-contaminated	4	22	26	15.4
Contaminated	5	10	15	33.3
Dirty	6	4	10	60
Duration < 1 hr	1	17	18	5.6
1–2 hrs	6	34	40	15
> 2 hrs	9	3	12	75
Preop stay < 24 hrs	3	25	28	10.7
1–3 days	4	22	26	15.4
>3 days	9	7	16	56.3
Prophylaxis given	12	50	62	19.4
No prophylaxis	4	4	8	50
Shaving < 2 hrs	3	31	34	8.8
Night before	7	14	21	33.3
> 24 hrs before	6	9	15	40

Redness, swelling, and pain were the most frequently observed signs in patients who developed SSIs. Purulent discharge was seen in 75%, underscoring its diagnostic significance. *E. coli *was the most common pathogen isolated from SSI sites, followed by *S. aureus* and *Klebsiella pneumoniae*. Most isolates demonstrated sensitivity to higher-generation antibiotics, indicating possible resistance to first-line prophylactic agents. Most SSIs were diagnosed between postoperative days four and six (37.5%), consistent with typical superficial infection onset timelines. Notably, nearly 19% were identified after day 10, reinforcing the importance of extended follow-up (Table 4).

**Table 4:** Clinical and microbiological profile

Category	n	%
Clinical signs		
Redness and swelling	14	87.5
Purulent discharge	12	75
Fever > 38°C	9	56.3
Wound gaping	6	37.5
Pain	13	81.3
Microbiology		
Escherichia coli	6	37.5
Staphylococcus aureus	4	25
Klebsiella pneumoniae	3	18.8
Pseudomonas aeruginosa	2	12.5
No growth	1	6.3
Day of detection		
Day 2–3	2	12.5
Day 4–6	6	37.5
Day 7–10	5	31.3
After Day 10	3	18.7

SSI rates were nearly three times higher in open surgeries (31%) compared to laparoscopic procedures (10.7%), supporting the protective effect of minimally invasive techniques against infection. Patients with SSIs had nearly double the length of hospital stay (mean: 11.8 days) compared to those without infections (mean: 5.6 days), indicating significant healthcare resource utilization due to SSIs (Table 5).

**Table 5:** Surgical outcomes SSI: surgical site infection

Variable	n	%/Mean
Surgical approach		
Open surgery	42	31.0% SSI
Laparoscopic	28	10.7% SSI
Length of stay		
SSI present	11.8	±2.4 days
SSI absent	5.6	±1.9 days

Duration of surgery, prolonged preoperative hospital stays, emergency procedures, contaminated wound class, and diabetes mellitus were all found to be statistically significant risk factors for SSI (p < 0.05) (Table 6).

**Table 6:** Chi-square analysis of risk factors significantly associated with SSI p < 0.05 is considered significant; SSI: surgical site infection

Risk factor	Chi-square (χ²)	p-value	Significance
Duration of surgery > 2 hrs	14.02	0.001	Not significant
Preoperative stay > 3 days	10.68	0.005	Significant
Emergency surgery	5.62	0.018	Significant
Wound class (contaminated/dirty)	11.21	0.002	Significant
Diabetes mellitus	4.25	0.039	Significant

Multivariate analysis revealed that prolonged surgery (> two hrs), dirty wound class, extended preoperative stay, and diabetes were independent predictors of SSI. These findings align with existing literature and highlight modifiable targets for intervention (Table 7).

**Table 7:** Multivariate logistic regression (independent predictors of SSI) SSI: surgical site infection

Risk factor	Adjusted OR	95% CI	p-value	T-value
Duration of surgery > 2 hrs	5.72	1.76–18.64	0.003	2.9
Dirty wound class	4.39	1.29–14.94	0.018	2.37
Preoperative stay > 3 days	3.91	1.14–13.49	0.03	2.16
Diabetes mellitus	2.85	1.01–8.08	0.048	1.97

## Discussion

The demographic profile of patients in the present study, as shown in Table 1, reveals a predominance of individuals in the 31-40-year age group (25.7%), with a gradual decline in representation in older age brackets. This relatively younger population may be reflective of the hospital's surgical intake or regional health-seeking behavior patterns. While age was not found to be a statistically significant factor for SSI within this sample, other studies suggest that advancing age is indeed a well-established risk factor. In a prospective observational study on colorectal surgeries, Panos et al. observed that patients over 70 years of age had significantly higher SSI incidence (p = 0.033), which was attributed to age-related physiological changes, immunosenescence, and the higher likelihood of comorbidities and frailty in older individuals [11]. Advanced age is an independent risk factor for SSIs due to delayed healing and poor blood flow. While gender alone is not always significant, male patients may have a higher risk due to differences in skin flora and lifestyle behaviors.

Patients with higher BMIs, particularly those who are overweight or obese, are at increased risk for SSIs. This is due to impaired tissue oxygenation and wound healing, making BMI a crucial, actionable factor in preoperative risk assessment. The negative impact of elevated BMI on surgical outcomes has led to the inclusion of BMI thresholds in several SSI risk prediction models, including those used in orthopedic and gastrointestinal procedures [12]. These insights underscore the need for preoperative weight optimization, where feasible, as part of a multi-modal infection prevention strategy.

The relationship between diabetes and SSIs is well-documented. Hyperglycemia impairs neutrophil chemotaxis, reduces phagocytic activity, and disrupts collagen synthesis, all of which are vital for wound healing. In the present study, patients with diabetes were found to be disproportionately affected by SSIs, mirroring trends observed globally. A large cohort study by Alkaaki et al. confirmed the association between diabetes and increased SSI risk in abdominal surgeries, reporting significantly higher infection rates among diabetic patients and highlighting their poor response to first-line prophylactic antibiotics due to the predominance of multidrug-resistant organisms such as extended-spectrum beta-lactamase (ESBL)-producing *E. coli *and *Enterococcus spp.* [13].

Other comorbidities, such as hypertension and chronic steroid use, though less consistently associated with SSIs in isolation, may contribute synergistically to impaired host defenses. In the study by Aga et al., immunosuppressive therapy, including steroids, was found to significantly elevate SSI risk, and this risk was further amplified in patients undergoing emergency surgeries or those with dirty wounds [14]. Comorbidity assessment should form part of routine preoperative risk stratification.

Lifestyle factors and their contribution to surgical site infection

Lifestyle factors like smoking and alcohol use are significant, often-overlooked risk factors for SSIs. Addressing these and other modifiable factors, such as BMI and diabetes control, through preoperative counseling is crucial for improving surgical outcomes.

Surgical procedure type and urgency as risk factors

In this study, appendectomy (22.9%) and laparotomy (20.0%) emerged as the most commonly performed abdominal procedures. Notably, 45.7% of surgeries were performed as emergencies, while the remainder were elective.

The distinction between elective and emergency surgeries is highly relevant in the context of SSI risk. Emergency surgeries, by nature, allow less time for preoperative optimization, including glycemic control, correction of anemia, bowel preparation, or prophylactic antibiotic timing. In a prospective study by Alkaaki et al., emergency abdominal surgeries were significantly associated with a higher SSI incidence (35% in open emergency cases) compared to elective laparoscopic procedures (4%) [13]. Similarly, Panos et al. found that wound contamination and urgency were strongly associated with infection rates after colorectal surgery [11].

Large database studies from England and Wales also showed significantly reduced mortality and SSI incidence in laparoscopic emergency surgeries compared to open procedures (mortality 6.0% vs. 9.1%; SSI data not isolated, but hospital stay and infection proxies were improved). These findings reinforce the notion that both the type and timing of surgery are key SSI determinants, with emergency open procedures requiring focused infection prevention measures.

Wound class and infection risk

The current study demonstrates a clear escalation in SSI rates across wound contamination levels. Clean wounds had an SSI rate of 5.3%, whereas dirty wounds showed a staggering 60% infection rate, confirming the validity of CDC wound classification as a predictive tool. The direct correlation between wound class and SSI risk is strongly supported by international evidence.

A large multicenter trial by Mihaljevic et al. found that contaminated abdominal wounds had a nearly threefold higher SSI risk than clean-contaminated ones. Their meta-analysis of 3,695 patients showed a risk ratio of 0.44 for contaminated wounds when wound protectors were used, indicating that intervention at the level of exposure can mitigate infection burden even in high-risk cases [15].

Furthermore, a 2024 trial by Yoo et al. found that wound protectors significantly reduced SSI incidence (10.9% vs. 20.5%) in gastrointestinal surgeries, especially in clean-contaminated and contaminated procedures, lending further support to using such devices selectively based on wound class [16].

Our data support this evidence: contaminated wounds had a 33.3% SSI rate, while clean-contaminated cases had 15.4%. These findings highlight the importance of tailoring preventive interventions to the wound category.

Duration of surgery and surgical site infection

This study’s findings reinforce that prolonged operative duration is a significant independent risk factor for SSI, with infection rates rising from 5.6% for surgeries < one hour to 75.0% for surgeries lasting more than two hours. Extended operative time may reflect technical complexity, greater tissue manipulation, and increased exposure to environmental contaminants-factors conducive to infection. Alkaaki et al. reported similar findings in their cohort, where longer surgeries were independently associated with a higher risk of SSI, especially in open abdominal operations [13]. 

Preoperative hospital stay and its association with surgical site infection

Preoperative hospital stays over three days significantly increased SSI rates to 56.3%, compared to only 10.7% for patients admitted less than 24 hours prior. This highlights prolonged stays as a major risk factor for infection. In a multicenter study conducted by Li et al. (2021) on emergency abdominal surgeries in China, prolonged hospital stay prior to surgery was independently associated with higher SSI risk [12]. The rationale is multifactorial: extended stays increase exposure to hospital flora, contribute to delays in definitive treatment, and often reflect systemic comorbidities or ongoing infection. Similarly, Aga et al. (2015) found that preoperative hospitalization > 48 hours significantly predicted infection, particularly in patients undergoing biliary and gastrointestinal surgeries [14]. Preoperative delays in elective cases should be minimized to reduce exposure to resistant hospital pathogens.

Antibiotic prophylaxis and surgical site infection prevention

Administering prophylactic antibiotics lowered the SSI rate from 50% to 19.4%. This reinforces the critical importance of timely antibiotic prophylaxis, adherence to global guidelines, and using local antibiograms to prevent surgical infections. Alkaaki et al. reported that incorrect timing and suboptimal antibiotic choice led to increased incidence of SSI, especially in patients undergoing contaminated surgeries [13].

Timing of preoperative shaving and its impact on infection

Preoperative shaving over 24 hours before surgery significantly increases SSI risk, whereas shaving within two hours yields the lowest risk. These findings, consistent with global guidelines, reinforce the need for standardized practices using clippers immediately before surgery.

Clinical presentation of surgical site infection

Clinical signs in patients with SSI were typical and consistent with CDC definitions. As shown in Table 4, redness and swelling were present in 87.5%, purulent discharge in 75%, and fever in 56.3% of cases. These signs reflect both superficial and early deep incisional involvement.

This presentation aligns with the findings of Astagneau et al. (2001), who described pain, erythema, and discharge as the triad of early indicators in incisional SSIs [17]. In a more recent prospective study by Panos et al., most SSIs manifested as local symptoms before systemic signs, supporting routine postoperative wound inspection from day 2 onwards [11].

Microbiological profile of surgical site infection pathogens

The microbiological spectrum of pathogens isolated in this study predominantly included Gram-negative organisms, with *Escherichia coli *(37.5%), *Staphylococcus aureus* (25%), and *Klebsiella pneumoniae *(18.8%) being the leading causative agents. This is in line with findings from numerous Indian and international studies.

Alkaaki et al. reported that *E. coli* and *Enterococcus spp*. were the most common isolates, and over 60% of these showed resistance to standard prophylactic antibiotics like cefazolin [13]. Similar trends were documented in the systematic review by Marzoug et al. (2023), where *E. coli *was consistently dominant, especially in bowel and perforation-related surgeries [18].

The rise of methicillin-resistant *Staphylococcus aureus *and ESBL organisms in surgical wounds highlights the need for antimicrobial stewardship. Tackling modifiable risks like prolonged hospital stays and inadequate prophylaxis is crucial for reducing SSIs.

Timing of surgical site infection presentation in the postoperative period

Most SSIs appeared between postoperative days four and 10, though a portion emerged later. This finding emphasizes the need for continued post-discharge surveillance, like outpatient wound checks, to ensure timely diagnosis and accurate reporting.

Impact of surgical technique on surgical site infection risk

Open surgery had a significantly higher SSI rate (31.0%) compared to laparoscopic procedures (10.7%). This validates that less invasive techniques lead to fewer infections, quicker recovery, and shorter hospital stays, advocating for their use when possible.

Length of hospital stay in relation to surgical site infection

Patients with SSIs had an average hospital stay of 11.8 days, nearly double that of uninfected patients. This finding confirms that SSIs significantly extend hospital stays, increasing both patient morbidity and healthcare costs.

Risk factor analysis confirmed that prolonged surgery, wound contamination, emergency status, extended preoperative stay, and diabetes significantly predicted SSI, consistent with existing literature.

Study limitations

This study has certain limitations that should be acknowledged. First, the relatively small sample size from a single tertiary care center may limit the generalizability of the findings to other populations and healthcare settings. Second, the cross-sectional design restricted the ability to establish temporal or causal relationships between risk factors and the development of SSIs. Third, microbial culture and sensitivity testing were performed only on clinically suspected cases of SSI, which may have led to underestimation of subclinical infections. Additionally, factors such as perioperative nutritional status, detailed glycemic control, and surgeon-specific practices were not comprehensively assessed, which could have influenced outcomes. Future multicenter prospective studies with larger cohorts are warranted to validate these findings and explore additional modifiable determinants.

## Conclusions

SSIs following abdominal surgery remain a preventable but significant complication, associated with prolonged hospital stay and increased healthcare costs. Independent predictors identified in this study include prolonged operative duration, dirty wound classification, extended preoperative hospitalization, and diabetes. Strengthening infection control strategies through timely prophylaxis, strict aseptic technique, antimicrobial stewardship, and wider adoption of minimally invasive approaches can substantially reduce SSI rates. These findings emphasize the importance of targeted, context-specific interventions in tertiary care hospitals.

## Appendices

Appendix 1

**Table 8:** Questionnaire part 1 (demographic details)

Name			
1. Age in years			
2. Gender:	Male	Female	Other
3. Weight in kg			
4. Height: in cm			
5. BMI (calculated): in kg/m²			
6. Occupation:			
7. Address (for follow-up):			
8. Contact number:			

**Table 9:** Questionnaire part 2 (clinical profile)

Comorbidities	☐ Diabetes mellitus	☐ Hypertension	☐ Malignancy	☐ Others (Specify): ____________________________
9. Lifestyle history				
a. Smoking	☐ Yes	☐ No		
b. Alcohol consumption	☐ Yes	☐ No		
10. Nutritional status:				
a. Recent weight loss	☐ Yes	☐ No		
b. Dietary deficiency suspected	☐ Yes	☐ No		

**Table 10:** Questionnaire part 3 (preoperative factors)

Type of surgery	☐ Elective	☐ Emergency
11. Preoperative hospital stay		
12. Shaving done:	☐ Yes	☐ No
13. Preoperative antibiotic given	☐ Yes	☐ No
14. Antibiotic used		

**Table 11:** Questionnaire part 4 (intraoperative details)

Procedure name				
15. Surgical approach	☐ Open	☐ Laparoscopic		
16. Duration of surgery: in minutes				
17. Wound classification:	☐ Clean	☐ Clean-contaminated	☐ Contaminated	☐ Dirty/Infected
18. Drain used:	☐ Yes	☐ No		

**Table 12:** Questionnaire part 5 (postoperative follow-up) SSI: surgical site infection

Signs of SSI observed:	☐ Redness	☐ Swelling	☐ Pain	☐ Fever (>38°C)	☐ Discharge/Pus	☐ Wound gaping
Postoperative day SSI Suspected on which day						
19. Swab sent for culture	☐ Yes	☐ No				
a. Organism isolated						
b. Antibiotic sensitivity						
20. Antibiotics used for treatment						
21. Length of postoperative stay in days						
22. Readmission for SSI	☐ Yes	☐ No				
23. Wound Status at Follow-up (Day 10 or Suture Removal):						
